# The effect of remimazolam and propofol on post-hysteroscopy sleep quality under general anesthesia: a *post hoc* analysis

**DOI:** 10.3389/fphar.2025.1618190

**Published:** 2025-07-08

**Authors:** Chen Yang, Le Zhang, Yan Cheng, Jianying Hu, Yuyan Nie, Shen Sun, Shaoqiang Huang

**Affiliations:** ^1^ Department of Anesthesiology, Obstetrics and Gynecology Hospital, Fudan University, Shanghai, China; ^2^ Department of Anesthesiology, Shanghai Fifth People’s Hospital, Fudan University, Shanghai, China

**Keywords:** remimazolam, fatigue scale, post-hysteroscopy sleep quality, *post hoc* analysis, crossover trial, hysteroscopic surgery

## Abstract

**Background:**

This *post hoc* analysis trial compared the impacts of remimazolam and propofol anesthesia on sleep quality and fatigue after hysteroscopic surgery.

**Methods:**

The *post hoc* analysis excluded patients with a Pittsburgh Sleep Quality Index (PSQI) score >15 or incomplete data. Preoperative PSQI scores were compared between patients receiving remimazolam and propofol. Intraoperative monitoring was conducted using the Modified Observer’s Assessment of Alertness/Sedation (MOAA/S) scale. Postoperative sleep and fatigue were assessed using the Athens Insomnia Scale (AIS) and Fatigue Scale-14 (FS-14) via WeChat questionnaires on the first and second postoperative days.

**Results:**

Fifteen patients were included in this *post hoc* analysis. No significant differences were observed in AIS scores on postoperative days 1 and 2. Compared to the remimazolam group, patients in the propofol group experienced more awakenings (1 [0, 2] vs. 2 [1, 3], p = 0.029) and poorer sleep quality (1 [0, 2] vs. 2 [1, 2], p = 0.043) on the first postoperative day. FS-14 scores indicated higher fatigue in the propofol group on the first postoperative day (5 [3, 8] vs. 3 [2, 6], p = 0.035) with no significant difference on the second day. No intraoperative awareness was reported, and anesthesia satisfaction was similar between the two groups.

**Conclusion:**

The *post hoc* analysis revealed that remimazolam reduced fatigue, particularly mental fatigue, on the first postoperative day compared to propofol in patients undergoing hysteroscopic surgery, despite no significant differences in AIS scores. Further research is needed to explore these effects in longer surgical procedures.

## 1 Introduction

Postoperative Sleep Disorders (PSD) refer to a clinical syndrome characterized by reduced nighttime sleep duration, disrupted sleep structure, and abnormal circadian rhythms following surgery (Luo et al., 2020). Postoperative Fatigue Syndrome (POFS) is subjectively felt fatigue after surgery, manifesting as persistent tiredness, weakness, and decreased concentration (Rubin and Hotopf, 2002). Both are closely related and commonly occur after various surgeries, significantly reducing the quality of postoperative recovery (Su and Wang, 2018). Studies have shown that patients experience decreased postoperative sleep quality (Lei et al., 2017) and increased fatigue scores after propofol anesthesia (Li et al., 2018). In recent years, a safe and effective new benzodiazepine sedative, remimazolam, has been widely used for anesthesia induction and maintenance. Unlike propofol, remimazolam’s metabolites are inactive, and its effects can be reversed by flumazenil (Sneyd and Rigby-Jones, 2020; Kilpatrick, 2021).

In our hospital, young patients with endometrial cancer or complex hyperplasia, aiming to preserve fertility, undergo a treatment protocol involving hysteroscopic evaluation and resection combined with progestin therapy (Guan and Chen, 2022). We enrolled patients who underwent two hysteroscopies within a 3-month period, randomly assigning them to remimazolam or propofol anesthesia in a crossover study (Yang et al., 2024).

Given the scarcity of studies on the effects of remimazolam on postoperative sleep and fatigue, both domestically and internationally, the impact of remimazolam on these outcomes remains unclear. This study aims to address this knowledge gap by comparing the effects of remimazolam and propofol on postoperative sleep and fatigue in patients undergoing hysteroscopic surgery.This *post hoc* analysis trial compared the effects of remimazolam and propofol on the postoperative sleep and fatigue status of patients undergoing hysteroscopic surgery.

## 2 Methods

### 2.1 Design

This article documents a *post hoc* analysis of a previous single-blind, randomized crossover trial, which showed that BIS was higher and hemodynamic fluctuation was less in patients with ramazolam anesthesia compared with propofol anesthesia.Details on interventions can be found elsewhere (Yang et al., 2024). Briefly, the inclusion criteria were patients aged 18–40 years with a body mass index (BMI) of 20–28 kg/m^2^, scheduled for hysteroscopic evaluation and resection combined with progestin therapy for endometrial cancer or complex hyperplasia. On the day before surgery, After obtaining informed consent, the anesthesiologist responsible for the study added eligible patients to WeChat, distributed the Pittsburgh Sleep Quality Index (PSQI) questionnaire, (Zak et al., 2022), random numbers were generated using a random number generator software, and the pre-prepared sealed envelope marked with the corresponding number was opened, and patients were regimened according to the information inside the envelopes, marking the We Chat contacts with the regimen information. Depending on the anesthetic regimen for hysteroscopy, patients were randomly assigned to two regimens: one regimen received propofol first, followed by remimazolam anesthesia 3 months later; the other regimen was treated with remimazolam first, followed by propofol anesthesia 3 months later.

### 2.2 Samples and settings

Assuming a standard deviation of 10 for BIS values between groups when sedation scores are ≤1, and aiming to detect a mean BIS difference of ≥15, a paired t-test with α = 0.05 and power of 85% requires a sample size of 14. Given the 3-month study duration and an estimated attrition rate of 40%, we planned to enroll 20 patients.A total of 17 patients completed the randomized crossover study. In the *post hoc* analysis, the withdrawal criteria were those with PSQI >15 or incomplete postoperative data.

Upon admission to the operating room, routine non-invasive monitoring of blood pressure (Mean Arterial Pressure, MAP), heart rate (HR), and oxygen saturation (SPO2) was commenced. Anesthesia induction (T0) involved TCI pump-administered remifentanil (Ce 1.58 ng/mL). Concurrently, patients received either a slow injection of remimazolam (0.27 mg/kg over 30s, followed by a continuous infusion at 1 mg/kg/h followed by a continuous infusion at 1 mg/kg/h) or propofol (2.0 mg/kg over 30s, followed by a continuous infusion at 6 mg/kg/h). The Modified Observer’s Assessment of Alertness/Sedation Scale (MOAA/S) (Pastis et al., 2022) was assessed every minute after administration. T1 was recorded when MOAA/S fell below 2. T2, T3, and T4 marked the insertion of the laryngeal mask, the start of hysteroscopic cervical dilation, and surgery end, respectively. Patient awakening was denoted as T5. All patients were discharged on the same day,no postoperative analgesic protocol was implemented.

### 2.3 Data collection

Postoperatively, patients were surveyed at 8 a.m. on the first and second days via WeChat using the Athens Insomnia Scale (AIS) (Gómez-Benito et al., 2011) to assess sleep quality. The AIS includes eight items evaluating sleep onset, nocturnal awakenings, early morning awakening, total sleep time, overall sleep quality, daytime mood, daytime physical function (memory, cognitive ability, attention), and daytime somnolence. Each item is scored from 0 (none) to 3 (severe); a total score <4 indicates no sleep disturbance, 4–6 suggests possible insomnia, and >6 indicates sleep disturbance. The Fatigue Scale (FS-14) (Chalder et al., 1993) consisting of 14 items across two dimensions—physical and mental fatigue—was also administered. This binary scale scores from 0 to 1, with physical fatigue maxing at 8, mental fatigue at 6, and a total possible maximum of 14. A score ≥5 indicates fatigue syndrome. Intraoperative awareness was assessed on the first day postoperatively, and anesthesia satisfaction was evaluated on the second day postoperatively. Blinded anesthesia nurses recorded questionnaire data.

The primary outcome was AIS scores on the first postoperative day. Secondary outcomes included AIS scores on the second postoperative day and FS-14 scores on both the first and second postoperative days. Intraoperative hemodynamic data (MAP, HR, etc.) were re-evaluated.

### 2.4 Data analysis

Data analysis was conducted using SPSS 22.0. The Kolmogorov‒Smirnov test assessed univariate data distribution. Normal data are reported as mean ± standard deviation ( $\bar{x}$ ± s). The paired-sample t-test compared regimens, with Bonferroni correction for intra-group comparisons, setting significance at P < 0.0125. Non-normal data are presented as median (interquartile range), with the paired rank sum test used for inter-regimen comparisons. Count data, expressed as rates, were analyzed using the chi-squared test, with P < 0.05 indicating significance.

### 2.5 Ethical considerations

The original study (Yang et al., 2024) had been approved by the University’s Institutional Review Board (IRB2022-134), and all patients had provided informed written consent. The original trial was registered at the Chinese Clinical Trial Registry (ChiCTR2200064551, Principal investigator: Chen Yang, https://www.chictr.org.cn/showproj.html?proj=177003, Date of registration: 2022/10/11).

## 3 Results

**FIGURE 1:**
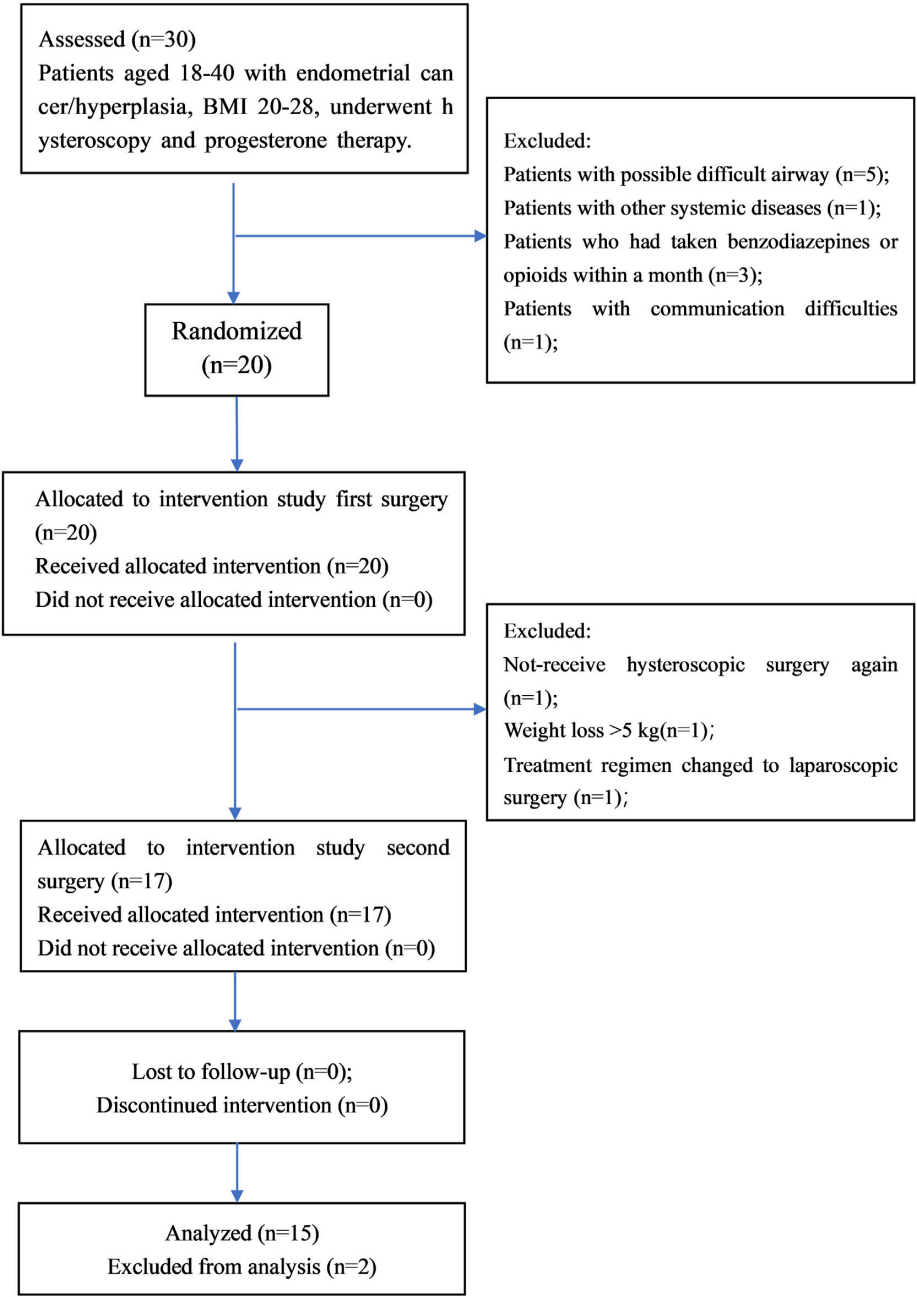
Flow chart.

There were 17 patients in the crossover trial, two were excluded due to incomplete follow-up data. Consequently, the final analysis was conducted on 15 patients (Figure 1). Of the 15 patients, mean age was 34.4 ± 5.8 years; height, 163.5 ± 7.3 cm; weight, 63.5 ± 11.7 kg; post-three-month weight, 63.9 ± 12.3 kg, with no significant weight change (P = 0.67). Intraoperatively MAP and HR were assessed (Table 1). Remimazolam induced no significant changes in MAP and HR from baseline. Propofol led to significant changes in MAP at T2 and T3, and HR at T2, T3, and T4 vs. baseline (p < 0.0125). Comparing regimens remimazolam resulted in higher HR at T2, T3, and T4 than propofol (p < 0.05), with no significant MAP differences between regimens.

**TABLE 1:** Comparison of hemodynamic parameters at different time points between the two regimens.

Time points	MAP		HR	
	Remimazolam N = 15	Propofol N = 15	Remimazolam N = 15	Propofol N = 15
T0	95.3 ± 12.7	97.5 ± 11.4	71.1 ± 12.2	71.6 ± 8.9
T1	88.9 ± 10.1	88.4 ± 10.6	72.6 ± 12.1	71.3 ± 12.5
T2	86.4 ± 13.4	85.7 ± 8.9*	73.5 ± 15.3^#^	64.2 ± 12.9*
T3	88.1 ± 13.1	84.2 ± 10.5*	72.0 ± 12.9^#^	66.2 ± 10.7*
T4	88.7 ± 9.1	87.1 ± 10.7	69.7 ± 14.2^#^	61.3 ± 13.2*

Values are presented as mean ± standard deviation.The paired-sample t-test compared regimens, with Bonferroni correction for intra-group comparisons, setting significance at P < 0.0125.

*Variations from baseline at T0 are statistically significant (P < 0.0125).

# Compared with propofol regimens are statistically significant (p < 0.05).

MAP: mean arterial pressure; HR: heart rate; T0:timepoint of anesthesia induction; T1:timepoint of MOAA/S ≤ 2; T2:timepoint of laryngeal mask insertion; T3:timepoint of the start of hysteroscopic cervical dilation; T4:timepoint of surgery end; T5:timepoint of patient awakening.

Preoperative PSQI scores were similar between regimens: 4 (3–5) vs. 4 (3–6), p = 0.377. Surgical durations were comparable. Postoperative AIS scores on days 1 and 2 were not significantly different between regimens (Table 2). However, careful analysis of the contents of AIS score showed that on the first postoperative night, patients in the propofol regimen had more night time awakenings (1 [0, 2] *versus* 2 [1, 3], *p* = 0.029) and poorer sleep quality (1 [0, 2] *versus* 2 [1, 2], p = 0.043) compared those in remimazolam regimen.

**TABLE 2:** Comparison of postoperative Athens Insomnia Scores between the two regimens.

Questionnaire content	Postoperative day 1					Postoperative day 2				
	Remimazolam N = 15	Propofol N = 15	Mean difference	95% CI of the difference	P	Remimazolam N = 15	Propofol N = 15	Mean difference	95% CI of the difference	P
Insomnia scoring	4 (1.5)	4 (2.6)	0.40	(-1.42, 0.62)	0.219	2 (1.3)	2 (1.4)	0.78	(-2.86, 0.39)	0.121
Time taken to fall asleep	0 (0.1)	0 (0.1)	0.02	(-0.13, 0.38)	0.568	0 (0.1)	0 (0.1)	0.04	(-0.32, 0.56)	0.562
Night time awakenings	1 (0.2)	2 (1.3)	0.34	(-0.03, 0.58)	0.029	0 (0.2)	1 (0.2)	0.25	(-0.11, 0.67)	0.063
Waking up earlier than desired	0 (0.1)	0 (0.2)	0.09	(-0.23, 0.31)	0.073	0 (0.1)	0 (0.1)	0.06	(-0.13, 0.31)	0.343
Total sleep duration	1 (1.2)	1 (0.2)	0.05	(-0.12, 0.11)	0.121	1 (0.2)	1 (0.1)	0.14	(-0.25, 0.32)	0.152
Overall sleep quality	1 (0.2)	2 (1.2)	0.07	(-0.06, 0.10)	0.043	1 (0.2)	1 (1.2)	0.20	(-0.14, 0.55)	0.078
Daytime physical functions	0 (0.1)	0 (0.1)	0.02	(-0.09, 0.28)	0.573	0 (0.1)	0 (0.1)	0.07	(-0.12, 0.25)	0.471
Daytime mood	0 (0.1)	1 (0.1)	0.12	(-0.11, 0.35)	0.073	0 (0.1)	1 (0.1)	0.12	(-0.09, 0.68)	0.067
Daytime sleepiness	0 (0.1)	0 (0.1)	0.08	(-0.45, 0.24)	0.452	0 (0.1)	0 (0.1)	0.06	(-0.13, 0.27)	0.473

Values are presented as median (interquartile range), with the paired rank sum test used for inter-regimen comparisons.

FS-14 scores, detailed in Table 3 showed higher fatigue on the first day in the propofol regimen (5 [3, 8] *versus* 3 [2, 6], p = 0.035). Mental fatigue was lower in the remimazolam regimen than those in the propofol regimen (1 [0, 2] *versus* 3 [2, 4], p = 0.023). Physical fatigue on second postoperative day was also lower than those in the propofol regimen (3 [0, 5] *versus* 4 [1, 6], p = 0.047). There was no intraoperative awareness, and anesthesia satisfaction was similar for both regimens (9 [8–10] vs. 9 [8–10], p = 0.78).

**TABLE 3:** Comparison of postoperative Fatigue Scores between the two regimens.

Questionnaire Content	Postoperative day 1					Postoperative day 2				
	Remimazolam N = 15	Propofol N = 15	Mean difference	95% CI of the difference	P	Remimazolam N = 15	Propofol N = 15	Mean difference	95% CI of the difference	P
Fatigue Score	3 (2.6)	5 (3.8)	0.51	(0.13, 0.25)	0.035	3 (1.6)	4 (2.6)	0.55	(-0.59, 1.78)	0.092
Physical Fatigue	3 (1.7)	4 (2.8)	0.27	(-0.03, 1.28)	0.052	3 (0.5)	4 (1.6)	0.66	(0.16, 2.16)	0.047
Mental Fatigue	1 (0.2)	3 (2.4)	0.65	(0.36, 0.81)	0.023	1 (0.2)	1 (0.2)	0.45	(-0.64, 1.21)	0.421

Values are presented as median (interquartile range), with the paired rank sum test used for inter-regimen comparisons.

## 4 Discussion

This *post hoc* analysis of previous single-blind, randomized crossover trial compared the effects of remimazolam and propofol on postoperative sleep and fatigue in patients undergoing hysteroscopic surgery. Additionally, physical fatigue scores were lower on postoperative day 2 in remimazolam regimen. However, no significant differences were observed in overall postoperative insomnia scores or anesthesia satisfaction between the regimens.

The study employed the PSQI scale to assess patients' sleep quality over the month preceding surgery. Considering that the PSQI may not accurately reflect short-term changes in sleep or the impact of specific events on sleep, (Zitser et al., 2022), the Athens Insomnia Self-report Questionnaire was used postoperatively to evaluate sleep changes more specifically, including nocturnal sleep patterns and daytime functioning. (Chung et al., 2011). Concurrently, the FS-14 scale was utilized to assess fatigue levels, offering a quantitative evaluation of both physical and mental fatigue with good internal consistency. (Nøstdahl et al., 2019). The study used WeChat push to collect postoperative 2-day scales. As patients were young women with a high acceptance of new media methods. WeChat is an effective way to build a communication platform between hospitals and patients. (Montag et al., 2018).

Sleep is a cyclical process, divided into NREM (non-rapid eye movement) sleep, typically constituting 75%–80% of the sleep cycle, and REM (rapid eye movement) sleep, characterized by heightened brain activity and dreaming. (Al et al., 2021). Compared with men, women experience unique physiological changes during puberty, pregnancy, the postpartum period, and perimenopause, which can predispose them to sleep disturbances. (Aksan and Dilbaz, 2024). Research has consistently demonstrated a higher prevalence of sleep disorders in women. (Van Someren, 2021). Postoperative sleep disturbances, including difficulty falling asleep, nighttime awakenings, and early morning awakenings, may arise from various factors such as surgical trauma, effects of anesthetic medications, pain, discomfort, and psychological stress. These disturbances can negatively impact patient recovery and rehabilitation. (Rosenberg-Adamsen et al., 1996). In this study, the majority of the participants were young women may further contribute to an increased risk of postoperative sleep disorders.

Numerous studies have shown that propofol disrupts the postoperative sleep-wake cycle by affecting melatonin secretion and the locus coeruleus-norepinephrine (LC-NE) system, leading to decreased sleep quality on the first postoperative day. (Li et al., 2018; Kushikata et al., 2016; Du et al., 2018). Additionally, propofol has been found to influence the levels of inflammatory mediators, such as IL-6 and IL-8, thereby affecting inflammatory pathways and contributing to the occurrence of postoperative sleep disturbances. (Sayed et al., 2015; Qiao et al., 2015). Propofol inhibits REM sleep but can prolong slow-wave sleep, increasing deep sleep duration. (Lewis et al., 2018). Benzodiazepines also alter sleep architecture by extending NREM sleep, which increases total sleep time but notably suppresses slow-wave and REM sleep. (de Mendonça et al., 2023). Intraperitoneal administration of midazolam in rats significantly increased NREM sleep after 6 h. (Lancel et al., 1997). The latest retrospective cohort study found that compared with propofol, remimazolam in elderly patients undergoing spinal surgery was associated with smaller changes in melatonin and cortisol concentrations. It is speculated that the smaller impact on sleep rhythm may help alleviate postoperative sleep disorders. (Yaqiu et al., 2025).

In elderly patients undergoing total joint arthroplasty, the use of remimazolam for intraoperative sedation did not significantly improve postoperative sleep quality as assessed by the Richards-Campbell Sleep Questionnaire (RCSQ). However, there was a slight increase in scores related to sleep recovery and overall sleep quality. (Deng et al., 2023). This study found no differences in overall postoperative AIS scores between the two regimens. The minor differences observed in the number of nocturnal awakenings and overall sleep quality may not be clinically significant, considering the mean differences and the range of the 95% confidence intervals.Despite using a randomized crossover method to minimize bias from population differences, considering the short duration of hysteroscopic surgery, averaging around 10 min with total anesthesia time not exceeding 20 min, it is unlikely to have a significant effect on postoperative insomnia index scores on the first and second days. The effects of longer surgeries warrant further investigation.

Previous studies have found that changes in HR after anesthesia reflect the excitability of the vagus nerve and the circulatory function of patients, and the low functional state of the cardiovascular system is significantly related to the occurrence of POFS (Yu et al., 2015). This study found smaller HR fluctuations after remimazolam induction compared to propofol, suggesting circulation stability in line with prior results (Ye et al., 2023). This might explain the lower fatigue scores on day 1 with remimazolam. Inflammatory responses are also tied to subjective postoperative fatigue (Späth-Schwalbe et al., 1998).

This study has some limitations. Firstly, it does not exclude the impact of opioid medications on postoperative sleep. Clinical reports indicate that although remifentanil does not inhibit nocturnal melatonin secretion, it significantly suppresses REM sleep in subjects (Cao and Javaheri, 2018; Cutrufello et al., 2020). The disruptive effect of opioids on sleep architecture is dose-dependent; however, for patients experiencing pain, the analgesic effect of opioids reduces awakenings during sleep and improves sleep efficiency (Kondili et al., 2012). Secondly, this study only involves data from 1 day before surgery to 2 days after surgery, monitoring short-term sleep and fatigue conditions in patients. Research shows that postoperative sleep-wake cycle disturbances are most evident from day 1 to day 2 after surgery and can persist until day 6 (van Zuylen et al., 2022). It remains unclear whether longer follow-up would yield positive findings. Lastly, this study used subjective scales widely employed both domestically and internationally. Although simple and practical, these scales are susceptible to emotional and environmental influences. Objective assessment methods, such as using sleep monitors for EEG readings, wearable devices like smartwatches to track physiological parameters, and software to evaluate task completion speed and accuracy, are potential options for obtaining objective evaluation indices. However, these methods have limitations, particularly in requiring specialized equipment. Given that patients in this study were not hospitalized, implementing monitoring within 2 days post-operation was challenging. Future applications of these methods may be more feasible in studies involving hospitalized patients.

## 5 Conclusion

This *post hoc* analysis trial compared the effects of remimazolam and propofol anesthesia on postoperative sleep and fatigue status in patients undergoing hysteroscopic surgery. Although no differences were observed in the AIS scores over two postoperative days, patients in the remimazolam regimen exhibited lower levels of fatigue on postoperative day 1, particularly in terms of mental fatigue. This suggests that remimazolam may enhance early postoperative recovery quality in short-duration surgeries, making it a preferable choice for accelerating postoperative recovery compared to propofol. Future research is warranted to further compare the effects of propofol and remimazolam on fatigue levels and multidimensional evaluations of postoperative sleep quality in longer surgical procedures.

## Data Availability

The raw data supporting the conclusions of this article will be made available by the authors, without undue reservation.

## Abbreviations

PSQI, Pittsburgh Sleep Quality Index; AIS, Athens Insomnia Scale; FS-14, Fatigue Scale; MAP, mean arterial pressure; HR, heart rate; NREM, non-rapid eye movement; REM, rapid eye movement.

## References

[B1] Aksan A. Dilbaz B. (2024). Sleep disorders in women: what should a gynecologist know? Geburtshilfe Frauenheilkd 84 (11), 1043–1049. Published 2024 Aug 15. 10.1055/a-2371-0763 39524035 PMC11543111

[B2] Albqoor M. A. Shaheen A. M. (2021). Sleep quality, sleep latency, and sleep duration: a national comparative study of university students in Jordan. Sleep. Breath. 25 (2), 1147–1154. 10.1007/s11325-020-02188-w 33034880

[B3] Cao M. Javaheri S. (2018). Effects of chronic opioid use on sleep and wake. Sleep. Med. Clin. 13 (2), 271–281. 10.1016/j.jsmc.2018.02.002 29759277

[B4] Chalder T. Berelowitz G. Pawlikowska T. Watts L. Wessely S. Wright D. (1993). Development of a fatigue scale. J. Psychosom. Res. 37 (2), 147–153. 10.1016/0022-3999(93)90081-p 8463991

[B5] Chung K. F. Kan K. K. Yeung W. F. (2011). Assessing insomnia in adolescents: comparison of insomnia severity index, Athens insomnia scale and sleep quality index. Sleep. Med. 12 (5), 463–470. 10.1016/j.sleep.2010.09.019 21493134

[B6] Cutrufello N. J. Ianus V. D. Rowley J. A. (2020). Opioids and sleep. Curr. OpinPulm Med. 26 (6), 634–641. 10.1097/MCP.0000000000000733 32925368

[B7] de Mendonça F. M. R. de Mendonça GPRR Souza L. C. Galvão L. P. Paiva H. S. de Azevedo Marques Périco C. (2023). Benzodiazepines and sleep architecture: a systematic review. CNS Neurol. Disord. Drug Targets 22 (2), 172–179. 10.2174/1871527320666210618103344 34145997

[B8] Deng C. M. Meng Z. T. Yang J. Zhang C. J. Lu M. Wang Y. X. (2023). Effect of intraoperative remimazolam on postoperative sleep quality in elderly patients after total joint arthroplasty: a randomized control trial. J. Anesth. 37 (4), 511–521. 10.1007/s00540-023-03193-5 37055671 PMC10390348

[B9] Du W. J. Zhang R. W. Li J. Zhang B. B. Peng X. L. Cao S. (2018). The locus coeruleus modulates intravenous general anesthesia of zebrafish *via* a cooperative mechanism. Cell. Rep. 24 (12), 3146–3155. 10.1016/j.celrep.2018.08.046 30231998

[B10] Gómez-Benito J. Ruiz C. Guilera G. (2011). A Spanish version of the Athens insomnia scale. Qual. Life Res. 20 (6), 931–937. 10.1007/s11136-010-9827-x 21210225

[B11] Guan J. Chen X. J. (2022). The present status of metformin in fertility-preserving treatment in atypical endometrial hyperplasia and endometrioid endometrial cancer. Front. Endocrinol. (Lausanne) 13, 1041535. 10.3389/fendo.2022.1041535 36387903 PMC9646621

[B12] Kilpatrick G. J. (2021). Remimazolam: non-clinical and clinical profile of a new sedative/anesthetic agent. Front. Pharmacol. 12, 690875. 10.3389/fphar.2021.690875 34354587 PMC8329483

[B13] Kondili E. Alexopoulou C. Xirouchaki N. Georgopoulos D. (2012). Effects of propofol on sleep quality in mechanically ventilated critically ill patients: a physiological study. Intensive Care Med. 38 (10), 1640–1646. 10.1007/s00134-012-2623-z 22752356

[B14] Kushikata T. Sawada M. Niwa H. Kudo T. Kudo M. Tonosaki M. (2016). Ketamine and propofol have opposite effects on postanesthetic sleep architecture in rats: relevance to the endogenous sleep-wakefulness substances orexin and melanin-concentrating hormone. J. Anesth. 30 (3), 437–443. 10.1007/s00540-016-2161-x 26984688

[B15] Lancel M. Faulhaber J. Schiffelholz T. Mathias S. Deisz R. A. (1997). Muscimol and midazolam do not potentiate each other's effects on sleep EEG in the rat. J. Neurophysiol. 77 (3), 1624–1629. 10.1152/jn.1997.77.3.1624 9084625

[B16] Lei M. Zhang P. Liu Y. Fu F. Ye L. Zhu T. (2017). Propofol and sufentanil May affect the patients' sleep quality independently of the surgical stress response: a prospective nonrandomized controlled trial in 1033 patients' undergone diagnostic upper gastrointestinal endoscopy. BMC Anesthesiol. 17 (1), 53. 10.1186/s12871-017-0341-3 28359259 PMC5374607

[B17] Lewis S. R. Schofield-Robinson O. J. Alderson P. Smith A. F. (2018). Propofol for the promotion of sleep in adults in the intensive care unit. Cochrane Database Syst. Rev. 1 (1), CD012454. 10.1002/14651858.CD012454.pub2 29308828 PMC6353271

[B18] Li Y. Wang S. Pan C. Xue F. Xian J. Huang Y. (2018). Comparison of NREM sleep and intravenous sedation through local information processing and whole brain network to explore the mechanism of general anesthesia. PLoS One 13 (2), e0192358. 10.1371/journal.pone.0192358 29486001 PMC5828450

[B19] Luo M. Song B. Zhu J. (2020). Sleep disturbances after general anesthesia: current perspectives. Front. Neurol. 11, 629. 10.3389/fneur.2020.00629 32733363 PMC7360680

[B20] Montag C. Becker B. Gan C. (2018). The multipurpose application WeChat: a review on recent research. Front. Psychol. 9, 2247. 10.3389/fpsyg.2018.02247 30618894 PMC6297283

[B21] Nøstdahl T. Bernklev T. Fredheim O. M. Paddison J. S. Raeder J. (2019). Defining the cut-off point of clinically significant postoperative fatigue in three common fatigue scales. Qual. Life Res. 28 (4), 991–1003. 10.1007/s11136-018-2068-0 30506178

[B22] Pastis N. J. Hill N. T. Yarmus L. B. Schippers F. Imre M. Sohngen W. (2022). Correlation of vital signs and depth of sedation by modified observer's assessment of alertness and sedation (MOAA/S) scale in bronchoscopy. J. Bronchology Interv. Pulmonol. 29 (1), 54–61. 10.1097/LBR.0000000000000784 34238838

[B23] Qiao Y. Feng H. Zhao T. Yan H. Zhang H. Zhao X. (2015). Postoperative cognitive dysfunction after inhalational anesthesia in elderly patients undergoing major surgery: the influence of anesthetic technique, cerebral injury and systemic inflammation. BMC Anesthesiol. 15, 154. 10.1186/s12871-015-0130-9 26497059 PMC4619426

[B24] Rabelo F. A. Küpper D. S. Sander H. H. Fernandes R. M. Valera F. C. (2013). Polysomnographic evaluation of propofol-induced sleep in patients with respiratory sleep disorders and controls. Laryngoscope 123 (9), 2300–2305. 10.1002/lary.23664 23801248

[B25] Rosenberg-Adamsen S. Kehlet H. Dodds C. Rosenberg J. (1996). Postoperative sleep disturbances: mechanisms and clinical implications. Br. J. Anaesth. 76 (4), 552–559. 10.1093/bja/76.4.552 8652329

[B26] Rubin G. J. Hotopf M. (2002). Systematic review and meta-analysis of interventions for postoperative fatigue. Br. J. Surg. 89 (8), 971–984. 10.1046/j.1365-2168.2002.02138.x 12153621

[B27] Sayed S. Idriss N. K. Sayyedf H. G. Ashry A. A. Rafatt D. M. Mohamed A. O. (2015). Effects of propofol and isoflurane on haemodynamics and the inflammatory response in cardiopulmonary bypass surgery. Br. J. Biomed. Sci. 72 (3), 93–101. 10.1080/09674845.2015.11666803 26510263

[B28] Sneyd J. R. Rigby-Jones A. E. (2020). Remimazolam for anaesthesia or sedation. Curr. OpinAnaesthesiol 33 (4), 506–511. 10.1097/ACO.0000000000000877 32530890

[B29] Späth-Schwalbe E. Hansen K. Schmidt F. Schrezenmeier H. Marshall L. Burger K. (1998). Acute effects of recombinant human interleukin-6 on endocrine and central nervous sleep functions in healthy men. J. Clin. Endocrinol. Metab. 83 (5), 1573–1579. 10.1210/jcem.83.5.4795 9589658

[B30] Su X. Wang D. X. (2018). Improve postoperative sleep: what can we do? Curr. OpinAnaesthesiol 31 (1), 83–88. 10.1097/ACO.0000000000000538 PMC5768217 29120927

[B31] Van Someren E. J. W. (2021). Brain mechanisms of insomnia: new perspectives on causes and consequences. Physiol. Rev. 101 (3), 995–1046. 10.1152/physrev.00046.2019 32790576

[B32] van Zuylen M. L. Meewisse A. J. G. Ten Hoope W. Eshuis W. J. Hollmann M. W. Preckel B. (2022). Effects of surgery and general anaesthesia on sleep-wake timing: CLOCKS observational study. Anaesthesia 77 (1), 73–81. 10.1111/anae.15564 34418064 PMC9291940

[B33] Yang C. Jiao J. Nie Y. Shao W. Zhang H. Huang S. (2024). Comparison of the bispectral indices of patients receiving remimazolam and propofol for general anesthesia: a randomized crossover trial. Anaesth. Crit. Care Pain Med. 43 (3), 101377. 10.1016/j.accpm.2024.101377 38494158

[B34] Yaqiu L. Heng Z. Ruimin W. Xuri W. (2025). Effects of remimazolam and propofol on sleep rhythm and delirium after spinal surgery in elderly patients. Perioper. Med. (Lond). 14 (1), 18. 10.1186/s13741-025-00500-4 39934906 PMC11817572

[B35] Ye E. Wu K. Ye H. Zhang W. Chu L. Zhang K. (2023). Comparison of 95% effective dose of remimazolam besylate and propofol for gastroscopy sedation on older patients: a single-centre randomized controlled trial. Br. J. Clin. Pharmacol. 89 (11), 3401–3410. 10.1111/bcp.15839 37387195

[B36] Yu J. Zhuang C. L. Shao S. J. Liu S. Chen W. Z. Chen B. C. (2015). Risk factors for postoperative fatigue after gastrointestinal surgery. J. Surg. Res. 194 (1), 114–119. 10.1016/j.jss.2014.09.041 25450599

[B37] Zak R. S. Zitser J. Jones H. J. Gilliss C. L. Lee K. A. (2022). Sleep self-report and actigraphy measures in healthy midlife women: validity of the Pittsburgh sleep quality index. J. Womens Health (Larchmt) 31 (7), 965–973. 10.1089/jwh.2021.0328 35230171 PMC9299524

[B38] Zitser J. Allen I. E. Falgàs N. Le M. M. Neylan T. C. Kramer J. H. (2022). Pittsburgh sleep quality index (PSQI) responses are modulated by total sleep time and wake after sleep onset in healthy older adults. PLoS One 17 (6), e0270095. 10.1371/journal.pone.0270095 35749529 PMC9232154

